# *JAK2* V617F-positive essential thrombocythemia with subsequent development of immune thrombocytopenia: A case report

**DOI:** 10.1097/MD.0000000000017766

**Published:** 2019-11-01

**Authors:** Yasuhiro Oda, Shuku Sato, Emiko Kanbe, Wataru Kamata, Satomi Okada, Yotaro Tamai

**Affiliations:** Department of Hematology, Shonan Kamakura General Hospital, Kamakura, Kanagawa 247-8533, Japan.

**Keywords:** essential thrombocythemia, hydroxycarbamide, immune thrombocytopenia, *JAK2* V617F mutation, JAK-STAT signaling pathway, regulatory T cells, T helper type 17

## Abstract

**Rationale:**

Although essential thrombocythemia (ET) and immune thrombocytopenia (ITP) have different etiologies, 3 previous reports have described ET development in ITP patients, all of whom were positive for the *JAK2* V617F mutation. Here, we report the first published case of ITP following ET in the absence of other platelet disorders.

**Patient concerns:**

A 70-year-old woman with a five-year history of ET with *JAK2* V617F mutation treated with hydroxycarbamide for five months presented with petechiae on her limbs.

**Diagnosis:**

Her platelet count was 3 × 10^9^/L, with the immature platelet fraction being 29%. White blood cell count and hemoglobin level were normal. Bone marrow examination showed increased number of megakaryocytes, but no morphologic dysplasia in any lineage. G-band analysis revealed no abnormalities. Platelet transfusion and cessation of hydroxycarbamide did not affect the platelet count. Thrombocytopenia was unlikely to have been induced by drugs, heparin, systemic lupus erythematosus, or human immunodeficiency virus. Hence, a diagnosis of ITP was made.

**Interventions:**

The patient received oral prednisolone combined with intravenous immunoglobulin.

**Outcomes:**

Her platelet count rose to 310 × 10^9^/L and remained stable, while her steroid dose was reduced. Further blood tests revealed the presence of antibodies against *Helicobacter pylori*, and appropriate treatment was administered. Resumption of hydroxycarbamide did not induce thrombocytopenia.

**Lessons:**

Although ET and ITP have different etiologies, chronic inflammation and immune deregulation underlie both and may play an important role in the progression from one to the other. Further research is warranted to understand the relationship between ET and ITP.

## 1 Introduction

Essential thrombocythemia (ET), along with polycythemia vera and primary myelofibrosis, is one of the Philadelphia-negative myeloproliferative neoplasms. Somatic mutation of *JAK2*, *CALR*, or *MPL* is found in 90% of the patients with ET.^[1]^ The World Health Organization (WHO) classification of myeloid neoplasms and acute leukemia uses the following criteria to diagnose ET: platelet count ≥ 450 × 10^9^/L, increase in bone marrow megakaryocyte counts but not in the counts of other lineages, presence of clonal markers, and not meeting the WHO criteria for other hematologic diseases, with a more detailed description.^[2]^

In contrast, immune thrombocytopenia (ITP) is an acquired immune disorder that causes low platelet count. Thrombocytopenia has multiple etiologies, including increased platelet destruction by antiplatelet antibodies,^[3]^ T cell-mediated destruction of platelets,^[4]^ and impaired megakaryocytopoiesis due to antiplatelet antibodies.^[5]^ ITP has been defined as an autoimmune disorder characterized by isolated thrombocytopenia with a peripheral blood platelet count < 100 × 10^9^/L. Per its definition, primary ITP does not have any other cause and does not involve any underlying disorder that may be associated with thrombocytopenia. In contrast, secondary ITP comprises all forms of immune-mediated thrombocytopenia except primary ITP.^[6]^

Although ET and ITP are different diseases with distinct etiologies, 3 previously published case reports describe the development of ET in patients with ITP.^[7–9]^ These reports have discussed the possible mechanism of ET development after ITP diagnosis. However, to our knowledge, ITP following ET in the absence of other platelet disorders has not been reported. Here, we report the first published case of ITP following ET in the absence of other platelet disorders.

## 2 Case report

A 65-year-old woman was found to have an elevated platelet count of 570 × 10^9^/L during a routine checkup. Bone marrow biopsy revealed an increased number of megakaryocytes with no abnormalities in other lineages. Given the low likelihood of reactive thrombocytosis, she was diagnosed with ET. Genetic tests later revealed the presence of a *JAK2* V617F mutation. No thrombotic complications were found. Aspirin 100 mg per day was started to prevent thrombotic events. She was also prescribed lansoprazole 15 mg per day to prevent gastric ulcers. Platelet counts remained stable at around 600 × 10^9^/L, until four years later, when the platelet level gradually started to increase and reached 1390 × 10^9^/L. Aspirin and lansoprazole were stopped, and hydroxycarbamide 500 mg per day was started. As a result, the platelet count decreased to 410 × 10^9^/L.

**Figure 1:**
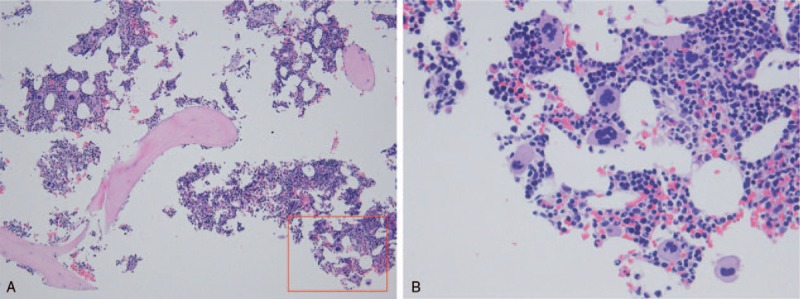
Histopathology of the bone marrow. Low-power view shows an increased number of megakaryocytes in mildly hyperplastic bone marrow with no fibrosis (panel A: hematoxylin and eosin (H&E) stain, original magnification ×100). High-power view reveals no morphologic dysplasia in any lineage including the megakaryocyte lineage and shows no blasts (panel B: H&E stain, original magnification ×400). The red square in panel A indicates the region shown in panel B.

**Figure 2:**
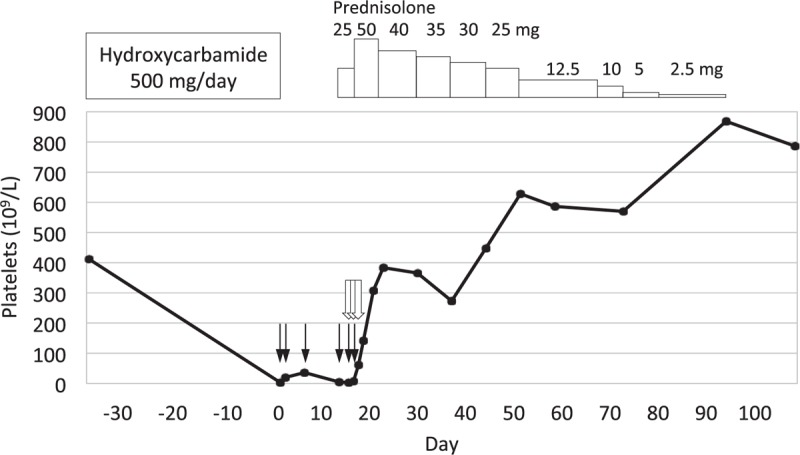
Platelet counts, medications, and transfusions. The first day of laboratory-proven thrombocytopenia is designated as day zero. One solid black arrow (↓) denotes 10 units of platelet transfusion (200 × 10^9^ platelets). One black arrow with white interior (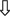) indicates 400 mg/kg of IVIg. Thrombocytopenia did not respond to platelets transfusions on day 0, 1, 5, and 12. Prednisolone 25 mg per day was started on day 12, but the platelet count was still low at 3 × 10^9^/L on day 14. A 3-day course of IVIg was started on day 14, additional platelets transfusions were given on day 14 and 15, and the prednisolone dosage was increased to 50 mg per day on day 15. The platelet counts started to increase on day 16.

Five months after hydroxycarbamide was started, the patient presented at our clinic with bruises on her limbs. On examination, petechiae were found on her oral mucosa and on her limbs. There were no signs of infection. Blood tests revealed a platelet count of 3 × 10^9^/L and an increased immature platelet fraction (IPF) of 29%. Her white blood cell count was 7.6 × 10^9^/L with a normal fraction, and her hemoglobin level was 13.7 g/dL. Schizocytes were absent. Bone marrow examination showed increased megakaryocyte counts, but no morphologic dysplasia in any lineage (Fig. 1). Hydroxycarbamide was stopped and platelet transfusions were administered at 10 units (200 × 10^9^ platelets) at a time, four times in two weeks. However, the platelet count remained low at 5 × 10^9^/L, suggesting that thrombocytopenia was caused by the destruction of platelets (Fig. 2). In addition, the anti-*Helicobacter pylori* antibody test gave positive results, whereas no anti-nuclear, anti-deoxyribonucleic acid (DNA), anti-double-stranded-DNA, anti-Smith, or anti-human immunodeficiency virus antibodies were detected. Overall, based on these results, ITP was suspected. Prednisolone 50 mg per day was started, and a 3-day course of intravenous immunoglobulin (IVIg) 400 mg/kg was administered. Treatment for *Helicobacter pylori* infection was administered simultaneously. Her platelet count immediately started to rise and reached 384 × 10^9^/L within a week. The prednisolone dosage was gradually decreased and tapered off after 2 and a half months, without recurrence of thrombocytopenia (Fig. 2).

Her platelet count gradually increased over time, and the patient reported increased bleeding tendency. Hydroxycarbamide was resumed ten months after its cessation at 500 mg every other day, after which the platelet count remains stable at around 500 × 10^9^/L.

## 3 Discussion

This report presents a case of ET followed by the development of ITP. Although differential diagnoses of thrombocytopenia initially included cytoreduction due to hydroxycarbamide, this could not explain the prolonged low platelet count observed after hydroxycarbamide cessation, increased number of megakaryocytes in the bone marrow, and unresponsiveness to multiple platelet transfusions. Increased megakaryocyte count in the bone marrow, increased IPF, and refractoriness to platelet transfusions suggested an etiology involving platelet destruction. Responsiveness to IVIg plus prednisolone and the absence of secondary causes of thrombocytopenia, except for *Helicobacter pylori* infection, led to a diagnosis of ITP.

ITP development in an ET patient is of particular interest. Considering the rarity of the two diseases, it is unlikely that they developed together by chance; the incidence of ET is 0.38 to 1.7/100,000 people per year,^[10,11]^ while the incidence of ITP is 3.3 to 3.9/100,000 people per year.^[12,13]^ Only 3 cases of the 2 conditions appearing together have been published to date. Huang et al described the case of a 14-year-old girl diagnosed with ITP followed by ET 3 years later.^[7]^ Sobas et al reported on a 45-year-old woman initially diagnosed with ITP who developed ET 21 years later.^[8]^ A recent report from Caocci et al described the case of a 72-year-old woman diagnosed with ITP who developed ET 13 years later.^[9]^ The patients in all these 3 cases were positive for the *JAK2* V617F mutation, which was also present in the case of ET with subsequent ITP development described in this report.

Although the etiologies of the 2 diseases seem different, chronic inflammation and immune deregulation are present in both.^[13,14]^*JAK2* V617F and other mutations in myeloproliferative neoplasms cause unregulated Janus kinase/signal transducer and activator of transcription (JAK-STAT) signaling.^[15]^ Panteli et al showed increased serum levels of interleukin (IL)-2 and soluble IL-2 receptor α in patients with ET, which may reflect chronic inflammation caused by increased lymphocyte activity.^[16]^*JAK2* V617F mutations constitutively activates *JAK2*, which in turn induces dysregulated phosphorylation of STAT3 and STAT5.^[17]^ The STAT3 pathway enhances leukocyte alkaline phosphatase expression and promotes inflammatory pathways, including nuclear factor-κB and IL-6–GP130–JAK pathways.^[18]^ STAT5 induces the cell proliferation responsible for the neoplastic character of ET.^[17]^

Meanwhile, ITP involves thrombocytopenia with different etiologies, including platelet destruction by antiplatelet antibodies,^[3]^ T cell-mediated destruction of platelets,^[4]^ and impaired megakaryocytopoiesis due to antiplatelet antibodies.^[5]^ A reduced number of regulatory T cells (Tregs) and their defective suppressive function have also been reported in ITP patients.^[19]^ Tregs play an important role in maintaining immunological self-tolerance, likely by secreting immunosuppressive cytokines and by suppressing, functionally modifying, and killing antigen presenting cells.^[20]^ An increasing body of evidence suggests that Treg impairment is a key pathogenetic mechanism of ITP.^[19,21]^ For instance, Stasi et al revealed that ITP patients who respond to rituximab also show a restoration in the number and function of Tregs.^[19]^

Treg impairment may cause deregulated immunosuppression. One previous study has shown that the level of IL-17A, an inflammatory cytokine produced by T helper type 17 (Th17) cells, is increased in patients with chronic immune thrombocytopenia.^[22]^ IL-17A induces the expression of pro-inflammatory cytokines (IL-6, tumor necrosis factor-α, and IL-1β).^[23]^ This may be relevant given the evidence from research on transgenic mice, where overexpression of retinoic acid-related orphan receptor γ-t (RORγt), a master regulator of Th17-cell development, led to polyclonal plasmacytosis and autoantibody production, likely via excessive IL-6 production due to IL-17.^[24]^ The transgenic mice also showed thrombocytopenia, anti-platelet antibodies in the serum, and increased number of megakaryocytes in the bone marrow,^[24]^ consistent with the etiology of ITP.

JAK-STAT pathway affects Treg and IL-17 expression. Yang et al showed that IL-6 upregulates expression of IL-23, which together with IL-6 activates STAT3 and hence promotes Th17 development. This differentiation was severely impaired in STAT3-deficient cells, which presented decreased expression of RORγt, reduced expression of IL-17, and increased levels of Tregs.^[25]^ This observation suggests that hyperactive STAT3 induces the overexpression of RORγt, increases the expression of Th17, and reduces the level of Tregs. André et al noted that major intracellular signaling pathways, including the JAK-STAT pathway, play a role in chronic activation of antigen presenting cells,^[26]^ which can escape suppression by Tregs and generate activated T cells that are refractory to the suppression by Tregs.^[27]^ Following these findings, one may hypothesize that constitutive activation of *JAK2* and its downstream pathway in ET may have contributed to the development of ITP in the case presented in this report. If this is the case, JAK inhibitors may theoretically help ameliorate ITP by suppressing the abnormal JAK-STAT signaling. Furthermore, one may also hypothesize that decreased immunological self-tolerance and deregulated immunosuppression due to impaired Tregs in ITP may have caused chronic inflammation and thus contributed to the development of ET in previously reported cases. Although these hypotheses are theoretical proposals based on insights from multiple basic research studies, they may provide a launch pad into investigations that elucidate the relationship between ET and ITP.

## 4 Conclusion

To our knowledge, this is the first reported case of a patient with ET and subsequent ITP development with no other platelet disorders. Although the etiology of these 2 diseases seem different, chronic inflammation and immune deregulation underlie both and may have played a key role in their concurrent development in this case and in previously reported cases. Further investigation is warranted to understand the relationship between ET and ITP.

## Author contributions

**Investigation:** Yasuhiro Oda, Shuku Sato, Emiko Kanbe, Wataru Kamata, Satomi Okada, Yotaro Tamai.

**Supervision:** Shuku Sato, Emiko Kanbe, Yotaro Tamai.

**Writing – original draft:** Yasuhiro Oda.

**Writing – review & editing:** Yasuhiro Oda, Yotaro Tamai.

Yasuhiro Oda orcid: 0000-0002-7043-7676.
